# Percolation may explain efficiency, robustness, and economy of the brain

**DOI:** 10.1162/netn_a_00246

**Published:** 2022-07-01

**Authors:** Yang Tian, Pei Sun

**Affiliations:** Department of Psychology and Tsinghua Laboratory of Brain and Intelligence, Tsinghua University, Beijing, China; Laboratory of Advanced Computing and Storage, Central Research Institute, 2012 Laboratories, Huawei Technologies Co. Ltd., Beijing, China

**Keywords:** Percolation, Brain connectivity, Excitation-inhibition balance, Information transmission efficiency, Robust flexibility, Brain economy

## Abstract

The brain consists of billions of neurons connected by ultra-dense synapses, showing remarkable efficiency, robust flexibility, and economy in information processing. It is generally believed that these advantageous properties are rooted in brain connectivity; however, direct evidence remains absent owing to technical limitations or theoretical vacancy. This research explores the origins of these properties in the largest yet brain connectome of the fruit fly. We reveal that functional connectivity formation in the brain can be explained by a percolation process controlled by synaptic excitation-inhibition (E/I) balance. By increasing the E/I balance gradually, we discover the emergence of these properties as byproducts of percolation transition when the E/I balance arrives at 3:7. As the E/I balance keeps increase, an optimal E/I balance 1:1 is unveiled to ensure these three properties simultaneously, consistent with previous in vitro experimental predictions. Once the E/I balance reaches over 3:2, an intrinsic limitation of these properties determined by static (anatomical) brain connectivity can be observed. Our work demonstrates that percolation, a universal characterization of critical phenomena and phase transitions, may serve as a window toward understanding the emergence of various brain properties.

## Author Summary

This research presents a novel framework to study functional connectivity on the largest yet brain connectome of the fruit fly, revealing that synaptic excitation-inhibition (E/I) balance characterizes the formation of dynamic brain connectivity as a percolation process. Various remarkable properties of brain functions, such as information transmission efficiency, robust flexibility, and economy, emerge as byproducts of percolation transition. These advantages can be simultaneously ensured at an optimal E/I balance 1:1, consistent with previous in vitro experimental predictions. Our work demonstrates percolation as a potential way to understand the emergence of brain function characteristics through connectivity.

## INTRODUCTION

To survive through the evolution, our brain should be efficient enough to process external information, robust to accidental damages (e.g., lesions), and economic in energy using. Over the last decades, this evolutionary inference has been corroborated by numerous neuroscience studies. The brain has been discovered to support highly efficient information transmission between neurons, circuits, and cortices, making it possible to promptly gather and distribute external information (Amico et al., 2021; Avena-Koenigsberger, Misic, & Sporns, 2018; Graham, Avena-Koenigsberger, & Misic, 2020; Mišić, Sporns, & McIntosh, 2014). Such information transmission efficiency, manifested as the low time cost of communications between neurons or high broadcasting capacity of information, is demonstrated to vary across different topological attributes of brain connectivity (Avena-Koenigsberger et al., 2018). Meanwhile, the brain is revealed to feature robust flexibility, a kind of capacity to tolerate the large-scale destruction of neurons or synaptic connections (e.g., by lesions) (Aerts, Fias, Caeyenberghs, & Marinazzo, 2016; Joyce, Hayasaka, & Laurienti, 2013; Kaiser, Martin, Andras, & Young, 2007) while maintaining robust brain functions (Achard, Salvador, Whitcher, Suckling, & Bullmore, 2006; Aerts et al., 2016; Alstott, Breakspear, Hagmann, Cammoun, & Sporns, 2009; Avena-Koenigsberger et al., 2017; Crossley et al., 2014; Joyce et al., 2013; Kaiser & Hilgetag, 2004; Kaiser et al., 2007). Although it inevitably requires a vast energy supply and occupies large space in the animal body due to the ultra-large neuron amounts, the brain is discovered to be economic in network wiring (low costs for embedding brain network into physics space) and network running (efficient in energy using) (Bullmore & Sporns, 2012; Friston, 2010; Hahn et al., 2015; Karbowski, 2007; Kiebel & Friston, 2011; Laughlin, van Steveninck, & Anderson, 1998; Sporns, 2011; Strelnikov, 2010). These economic properties are suggested as the functions of the brain network size, topology, and synaptic properties (Bullmore & Sporns, 2012).

Till now, it remains unclear where these remarkable properties of brain originate from. The close relationships between these properties and the brain network naturally lead to an emergentism hypothesis that argues these properties may originate from specific characteristics of brain connectivity. In the recent decades, abundant corollaries of this hypothesis have been verified from different perspectives. For instance, shortest paths in brain connectivity are inferred as a principal communication substrate in the brain according to the properties of information transmission efficiency (Avena-Koenigsberger et al., 2018). Although having a short average path is costly, real brain connectivity still possesses near-minimal path length (Betzel et al., 2016; Bullmore & Sporns, 2012; Kaiser & Hilgetag, 2006; Rubinov, Ypma, Watson, & Bullmore, 2015) in functional interactions (Goñi et al., 2014; Hermundstad et al., 2013) to support efficient information transmission. Moreover, brain connectivity is inferred as scale-free according to the robustness of scale-free networks (Albert, Jeong, & Barabási, 2000) and implied as small-world by the near-minimal path length (Bullmore & Sporns, 2012). While mammalian brains with scale-free connectivity are robust against random lesions, they are significantly vulnerable to hub-targeted attacks (Kaiser & Hilgetag, 2004; Kaiser et al., 2007). However, once the connectivity topology approaches a small-world network while maintaining scale-free property (e.g., the macroscopic human brain) (Achard et al., 2006; Alstott et al., 2009; Crossley et al., 2014; Joyce et al., 2013), the brain becomes more resilient to hub-targeted attacks than a comparable scale-free network and keeps equally robust to random attacks. Besides these mentioned corollaries, many other verified corollaries could be found, demonstrating that brain connectivity pattern critically shapes brain functions.

However, these corollaries alone are not sufficient for a complete demonstration of the emergentism hypothesis. Key supporting evidence remains absent because it is technically infeasible to capture and control the emergence of these properties *in vitro* or *vivo*, at least in the near future. One challenge arises from the scarcity of technology to record multi-mode (including both static and functional connectivity), fine-grained, and high-throughput connectome data (Sporns, Tononi, & Kötter, 2005). Another challenge is lacking experimental methods to modify the connectivity to control the emergence of these properties. Although the theoretical study may be an alternative choice, current classic models in neuroscience are either experiment driven and proposed for ex post facto analysis or simulation driven and designed for imitating phenomena rather than explaining mechanisms. These models are inapplicable for an assumption-free analysis when there is no experiment for reference. The absence of direct verification due to these challenges makes the validity of the hypothesis questionable.

Here we discuss the possibility of a feasible and physically fundamental demonstration of the emergentism hypothesis. These advantageous properties of brain functions are all relevant to the benefits or costs of forming specific functional connectivity (interactions among neurons) on the static connectivity (anatomical structure). Therefore, the emergence of these properties will be detectable if we can formalize the evolution of functional connectivity patterns on static connectivity. In the present study, we use a fine-grained and high-throughput brain connectome of the fruit fly, *Drosophila melanogaster* (Pipkin, 2020; Schlegel et al., 2021; Xu et al., 2020) to obtain precise information of static connectivity. As for functional connectivity, in real brains, it is subject to both static connectivity and the excitation-inhibition (E/I) properties of synapses; in our study, it is analyzed under an integrated framework: we begin with a massive neural dynamics computation (Gerstner, Kistler, Naud, & Paninski, 2014) on the whole brain (∼1.2 × 10^9^ times), enabling us to measure the coactivation probability of any pair of connected neurons and abstract static connectivity as a weight directed graph. Then, we formalize the formation of functional connectivity on the static connectivity, applying its equivalence relation with the percolation on random directed graphs, a universal characterization of critical phenomena and phase transitions (Dorogovtsev, Mendes, & Samukhin, 2001; Li et al., 2021). The motivation underlying this framework is to regulate the evolution of functional connectivity with a specific biological factor and verify whether these properties can be established as consequences of these manipulations. Limited by technology issues, the biological factor, the precise synaptic E/I information that neural dynamics computation requires, can not be recorded in the electron microscopy (EM) imagery of an insect brain yet (Xu et al., 2020). However, the E/I balance (i.e., the ratio between excitatory and inhibitory synapses) can act as a control parameter to randomize the E/I property of each synapse, offering an opportunity to verify if brain function properties emerge after specific percolation transitions.

## RESULTS

### Functional Connectivity Formation as Percolation

#### Topology properties of static connectivity.

**Figure 1.**
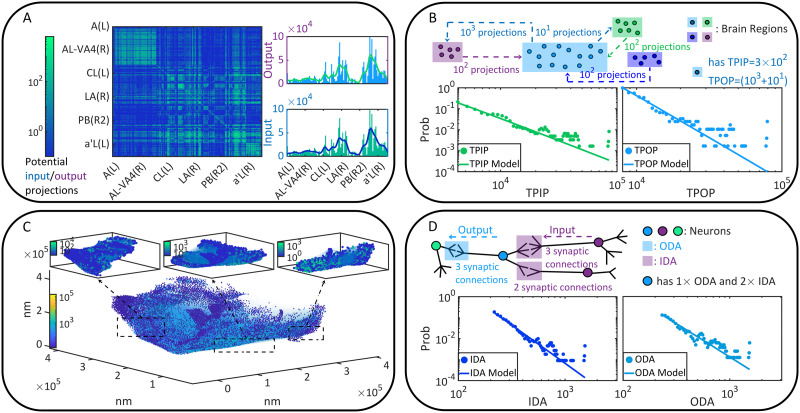
The static brain topology of the fruit fly, *Drosophila melanogaster*. (A) The macroscopic static connectivity, where potential input/output projections are counted between any two brain regions. Note that brain regions are sorted by their names. The heat map and histogram are shown in a coarse-grained way for a better vision. The heat map can be interpreted like an adjacent matrix, where the number of potential input/output projections of a brain region can be seen on the corresponding column (or row). (B) Variables of macroscopic static connectivity are graphically illustrated in this instance. The probability distributions of TPIP and TPOP are shown with corresponding estimated power law models. (C) The microscopic static connectivity, where each node represents a cell body and every directed edge represents a DA. Neurons are colored according to the number of involved synaptic connections. (D) Variables of microscopic static connectivity are graphically illustrated. The probability distributions of IDA and ODA are presented with estimated power law models.

Let us begin with the static or anatomical connectivity of the fruit fly brain. The data is acquired from the open source brain connectome lately released by FlyEM project (Xu et al., 2020). In the present study, neurons and synapses are considered only when the cell bodies are positioned precisely (assigned with a 10-nm spatial resolution coordinate). The selected data set, including 23,008 neurons, 4,967,364 synaptic connections (synaptic clefts), and 635,761 pairs of directionally adjacent relations (two neurons are directionally adjacent if one synaptic connection comes out of one neuron and leads to another), supports us to analyze static connectivity on the brain region or macroscopic scale (Figure 1A–B) and the cell or microscopic scale (Figure 1C–D). Please see the Materials and Methods for data acquisition.

The macroscopic static connectivity is analyzed in terms of the potential input projections or potential output projections that a brain region can receive from or cast to another brain region. These projections are treated as potential because static connectivity may not be equivalent to the functional one. In the analysis, we count these two kinds of projections between brain regions, based on which the total potential input projections (TPIP, the macroscopic in-degree) received by each region and the total potential output projections (TPOP, the macroscopic out-degree) coming from each region can be measured (Figure 1A). Please see the Materials and Methods and Figure 1B for variable descriptions.

In Figure 1B and Table 1, we analyze the power law distributions of TPIP and TPOP with a maximum likelihood estimation approach (R. Virkar & Clauset, 2014; Y. Virkar & Clauset, 2014), suggesting that the macroscopic static connectivity is plausibly scale-free (power law exponent *α* ∈ (2, 3) is estimated with ideal goodness). More details of power law analysis can be seen in Materials and Methods. Meanwhile, we verify the symmetry (e.g., with a balance between input and output projections) of macroscopic static connectivity using the Pearson correlations and the average change fractions (see Table 2). The connectivity is suggested as symmetric since (1) there are significant positive correlations between TPIP and TPOP (e.g., larger than 0.9); (2) The average change fraction of TPIP compared with TPOP is sufficiently small (e.g., smaller than 1). We also present other corroborative evidence derived from related variables to support these findings (please see Tables 5–6 and Figure 5 in Materials and Methods for additional information).

**Table 1.** Power law analysis results

Type	Variable	Probability distribution	Goodness of estimation	Scale-free or not
Macroscopic	TPIP	𝒫 (TPOP = *n*) ∝ *n*^−2.45^	0.0074	Yes
Macroscopic	TPOP	𝒫 (TPIP = *n*) ∝ *n*^−2.17^	0.0086	Yes
Microscopic	IDA	𝒫 (IDA = *n*) ∝ *n*^−3.69^	0.0348	No
Microscopic	ODA	𝒫 (ODA = *n*) ∝ *n*^−3.22^	0.0623	No

*Note*. Being scale-free requires 𝒫 ∝ *n*^−*α*^, where *α* ∈ (2, 3). Goodness of estimation is expected as to be less than 0.05.

**Table 2.** Symmetry analysis results

Variable	Variable	Pearson correlation	*p*	Average change fraction	Symmetric degree
TPIP	TPOP	0.9938	3.31667 × 10^−9^	$\frac{\left|\text{TPIP}-\text{TPOP}\right|}{\text{TPOP}}$ = 0.8215	Strictly strong
IDA	ODA	0.8817	< 10^−10^	$\frac{\left|\text{IDA}-\text{ODA}\right|}{\text{ODA}}$ = 0.6443	Less strong

*Note*. Strong symmetry requires a strong positive correlation (e.g., correlation > 0.9 and *p* < 10^−3^). Strong symmetry implies a small average change fraction (e.g., fraction < 1). The term “strictly strong” means that the strictest criterion of strong symmetry is completely satisfied. The term “less strong” means that the strictest criterion of strong symmetry is partly satisfied.

When turn to the cell scale, we characterize microscopic static connectivity depending on the directionally adjacent relation (DA) between neurons. Two neurons are directionally adjacent if there exists at least one synaptic connection coming from one of them to the other (see Figure 1C). Note that one DA may correspond to multiple synaptic connections because there can be more than one synaptic cleft. To offer an accurate characterization, we further subdivide variable DA according to input-output relations (e.g., pre- and postsynaptic relations). Specifically, we count input directionally adjacent relations (IDA) and the output directionally adjacent relations (ODA) for comparison on each neuron. Details of these variables are described in the Materials and Methods and Figure 1D.

In Figure 1D and Table 1, we show the power law analysis on the above defined variables. The same analysis is also conducted on other related variables (please see Table 3 and Figure 5 in Materials and Methods for additional information). Based on these results, the potential scale-free property of microscopic static connectivity is suggested as uncertain and nonrobust. Only a few variables (e.g., additional results in Table 5 in Materials and Methods) plausibly exhibit scale-free properties while others do not (e.g., results in Table 1). Meanwhile, symmetry analysis is applied to show the approximate symmetry of microscopic static connectivity. In Table 2, significant positive correlations and small average change fractions are observed between input-related and output-related variables. Similar properties can be seen on other variables of microscopic static connectivity (please see Table 6 in Materials and Methods for additional data). These results principally suggest that microscopic static connectivity is approximately symmetric, though the symmetric degree is not as strong as macroscopic static connectivity.

**Table 3.** Macroscopic variable definitions

Variable	Meaning
PIP	Potential input projections that a brain region can receive from another region
POP	Potential input projections that a brain region can cast to another region
TPIP	Total potential input projections that a brain region can receive from all other regions (macroscopic in-degree)
TPOP	Total potential input projections that a brain region can cast to all other regions (macroscopic out-degree)

The above analysis conveys three important messages: during the coarse-graining process from the cell scale to the brain-region scale, potential local asymmetry and diversity gradually fade away and eventually vanish owing to the loss of information in detailed connectivity. This finding encourages us to concentrate on fine-grained microscopic static connectivity in the subsequent analysis to control information loss. Meanwhile, although asymmetric upstream-downstream relations can be found among brain regions during information processing, these relations may merely exist in functional connectivity (static connectivity is principally symmetric). This finding reminds us that functional connectivity can not be simply reflected by static connectivity. Moreover, we speculate that the uncertain scale-free property is relevant with an existing controversy of whether static brain connectivity is scale-free or not (see pieces of supporting (Kaiser & Hilgetag, 2004; Kaiser et al., 2007)) and opposing evidence (Breskin, Soriano, Moses, & Tlusty, 2006; Humphries, Gurney, & Prescott, 2006). Scale-free property of the brain may critically rely on the granularity and the variables used in connectivity characterization.

To this point, the static connectivity of the fruit fly brain has been characterized, below we turn to formalize the formation of functional connectivity based on the static connectivity.

#### Neural dynamics computation and coactivation probability graph.

As discussed above, the fine-grained, high-throughput, and simultaneous recording of static connectivity and neural dynamics remains technically infeasible (Sporns et al., 2005). An alternative is to study the formation of functional connectivity based on the static connectivity through a theoretical way, whose first step is to analyze possible coactivation patterns among neurons. While one prerequisite of neural coactivation analysis, the static connectivity, has been obtained in the previous section, another prerequisite, the excitation-inhibition (E/I) properties of synapses, can not be recorded in the electron microscopy imagery of the insect brain (Xu et al., 2020). To avoid this obstacle, previous studies turn to mammalian brain regions (e.g., rat hippocampus) (Amini, 2010; Breskin et al., 2006; Cohen et al., 2010; Eckmann et al., 2007), where synaptic E/I properties could be easily recorded and controlled, but the connectivity imaging is much more coarse-grained and low-throughput.

We, however, treat this obstacle as an opportunity to study the role of synaptic E/I balance, a global factor that reflects the E/I properties of all synapses. Although precise synaptic E/I properties remain absent, they can be randomly assigned under the restriction of E/I balance *λ* ∈ (0, 1), the proportion of excitatory synapses in all synapses. After generating the static connection strength of every directionally adjacent relation *N*_*i*_ → *N*_*j*_ (here *N*_*i*_ and *N*_*j*_ are neurons), we can measure coactivation probability 𝒫_*ij*_ to define the dynamic connection strength. Here the coactivation probability is estimated by the leaky integrate-and-fire (LIF) model (Gerstner et al., 2014), a standard approach in neural dynamics computation. Please see the Materials and Methods for details.

**Figure 2.**
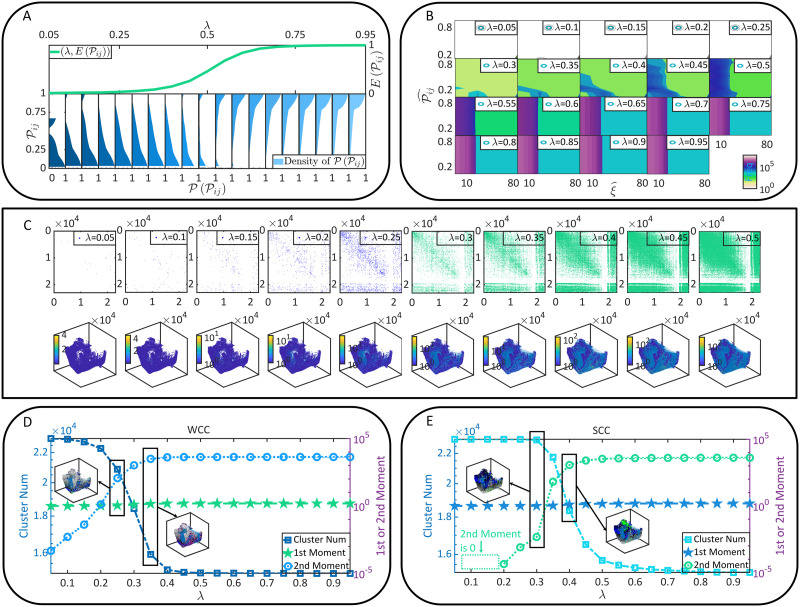
Neural dynamics and coactivation probability graphs. (A) The probability densities of 𝒫_*ij*_ under each *λ* condition are presented by colorful areas. Meanwhile, the first moment 𝔼(𝒫_*ij*_) is shown as a function of *λ*. (B), The observed occurrence frequency distributions of ($\hat{\xi }$, $\hat{𝒫}$_*ij*_) (counted on every directionally adjacent relation, DA) and how they vary with *λ*. (C) Instances of coactivation patterns under each *λ* condition are given (upper parallel). The coactivation of neurons *N*_*i*_ and *N*_*j*_ is represented as a point (*i*, *j*). Note that real coactivation patterns are much sparser than how they are displayed. Moreover, the implied functional connectivity by each coactivation pattern are shown (bottom parallel), where edges correspond to the DAs through which coactivation happens and neurons are colored based on the number of involved coactivation relations. (D–E) The cluster number as well as the first and second moments of cluster size are presented as functions of *λ*. Clusters are defined in terms of WCC (see D) and SCC (see E), respectively. Although the first moment of cluster size increases with *λ*, its variation is sufficiently slower than the second moment and therefore less visible. Missing data points in E are 0 in a logarithmic plot.

Letting *λ* vary from 0.05 to 0.95 (Δ*λ* = 0.05), we implement the above computation on every pair of *N*_*i*_ → *N*_*j*_ to obtain its dynamic connection strength in terms of coactivation probability 𝒫_*ij*_ under each *λ* condition. We treat 𝒫_*ij*_ as a variable and sample its probability density on the whole brain (Figure 2A). According to the density concentration tendency and the first moment 𝔼(𝒫_*ij*_), we suggest that 𝒫_*ij*_ increases with *λ* globally. This phenomenon can be double-checked if we analyze every binomial distribution *Bi*(𝒫_*ij*_) to find its peak value $\hat{𝒫}$_*ij*_ and the corresponding coordinate $\hat{\xi }$. The distribution is calculated in terms of a *ξ*-trial experiment (*ξ* = 100) where the success probability for each trial is 𝒫_*ij*_. Figure 2B illustrates the frequency distribution of ($\hat{\xi }$, $\hat{𝒫}$_*ij*_) sampled on the whole brain, revealing that $\hat{\xi }$ increases with *λ*. In Figure 2C, we show instances of coactivation patterns and their implied functional connectivity situations under each *λ* condition, which turn out to be denser when *λ* increases. Here the existence of coactivation between *N*_*i*_ and *N*_*j*_ is randomized following *Bi*(𝒫_*ij*_). A directed edge *N*_*i*_ → *N*_*j*_ is added to functional connectivity when *N*_*i*_ and *N*_*j*_ are coactivated. In Figure 2D–E, we analyze the properties of the weakly connected cluster (WCC) and the strongly connected cluster (SCC) (Bollobás, 2013) on the whole brain. It can be seen that the total numbers of WCCs and SCCs decline with *λ* because specific clusters become larger gradually (the first moment of cluster size maintains relatively constant while the second moment increases significantly). In sum, the computational costly coactivation analysis (∼1.2 × 10^9^ times of LIF model computation) enables us to study the E/I balance *λ* as a control parameter of functional connectivity formation. As expected, a higher E/I balance creates stronger functional connectivity because coactivation occurs more often. More neurons are included in the same cluster rather than maintain isolated, making it possible for large-scale communication between neurons to emerge.

However, the concordant increase of dynamic connection degree with the increase of E/I balance *λ* discussed above is insufficient to give a whole picture of all important information. A piece of missing information lies in that the observed formation of functional connectivity is a sigmoid-like process rather than a uniform growth process. While functional connectivity forms promptly when *λ* is relatively small, the formation speed becomes stable at a large *λ*. The nonuniform speed is not a trivial consequence of *λ* nor of an artificially manipulation. Therefore, we conjecture it as an emergence phenomenon triggered by *λ* and restricted by specific mechanisms.

#### Functional connectivity characterized by percolation.

**Figure 3.**
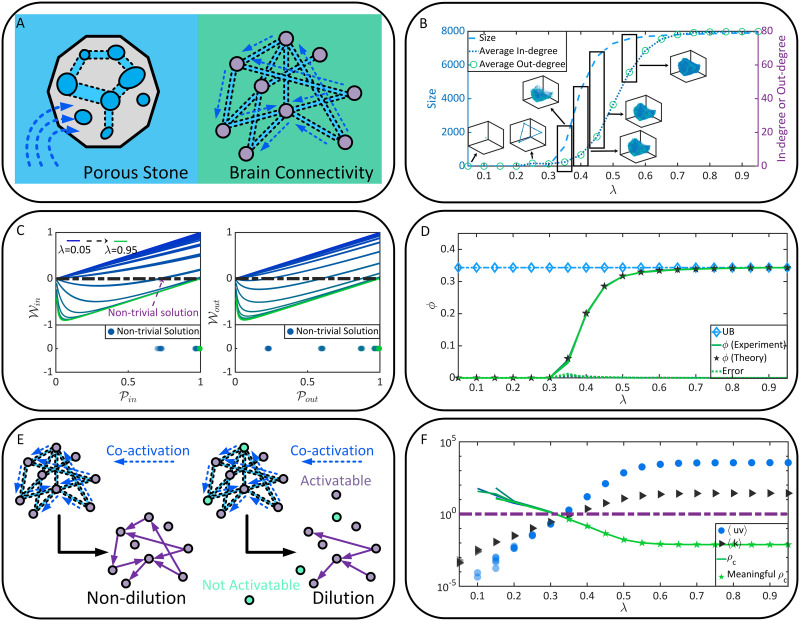
Functional connectivity formation as a percolation process. (A) The similarity between the soaking of porous stone and the functional connectivity of neurons. (B) The size, the average in-degree, and the average out-degree of the GSCC under different *λ* conditions. (C) 𝒫_*in*_ and 𝒫_*out*_ in brain connectome (upper parallel) and their nontrivial solutions (bottom parallel). (D) The probability *ϕ* predicted by Equation 7, the experimentally observed probability for a neuron to belong to the GSCC, and the upper bound (UB) of *ϕ* are presented as functions of *λ*. Meanwhile, the error between our theoretical predictions in Equation 7 and experimental observations is measured. (E) The difference between the nondilution percolation and the diluted one. (F) The percolation threshold *ρ*_*c*_, term 〈*uv*〉, and term 〈*k*〉 are shown as functions of *λ*. The meaningful values of threshold *ρ*_*c*_ are pointed out. Please note that D and F show *l* data sets of each variable independently derived from *l* (*l* = 5) times of functional connectivity generation (e.g., there are *l* sets of experimental data of *ϕ*). They highly overlap with each other to demonstrate that our observations are not accidental and keep consistency across different generated functional connectivity.

Let us step back from the above analysis and rethink the nature of functional connectivity. Functional connectivity is rooted in the coactivation probability between neurons and is affected by both static connectivity and the E/I balance *λ*. One can interpret functional connectivity as a communication pattern between neurons, where static connectivity serves as a network of information channels, and *λ* modifies the information transmission probability in these channels. Although this idea has been studied previously in neuroscience computationally (Amico et al., 2021; Graham et al., 2020; Shew, Yang, Yu, Roy, & Plenz, 2011), we discuss it from a more physically fundamental perspective—*percolation*. Percolation is a universal characterization of critical phenomena and phase transitions in a probabilistic form (Agliari, Cioli, & Guadagnini, 2011; Amini & Fountoulakis, 2014; Balogh & Pittel, 2007; Baxter, Dorogovtsev, Goltsev, & Mendes, 2010; Ben-Naim & Krapivsky, 2011; Callaway, Newman, Strogatz, & Watts, 2000; Dorogovtsev et al., 2001; Goltsev, Dorogovtsev, & Mendes, 2008; Li et al., 2021; Panagiotou, Spöhel, Steger, & Thomas, 2011; Radicchi & Fortunato, 2009). To understand percolation, one can imagine that a porous stone, where pores or tiny holes are connected randomly, is immersed in water (Figure 3A). Can the water come into the core or kernel of the stone? This question can be addressed by verifying the existence of specific paths connected between pores that run through the stone. It is trivial that the stone will be wetted thoroughly when the connection probability between pores is sufficiently large, as connected pores can form a cluster to penetrate the stone. Replacing the stone and pores by the brain and neurons, one can see the intriguing similarity between the soaking process of porous stone and the functional connectivity of neurons (Figure 3A). The only difference lies in that the space where connections can form changes from the lattice space of the stone to the random graph characterized by static connectivity. In decades, the equivalence relation between brain connectivity formation and the percolation on random graphs have attracted extensive explorations in biology (Bordier, Nicolini, & Bifone, 2017; Carvalho et al., 2020; Del Ferraro et al., 2018; Kozma & Puljic, 2015; Lucini, Del Ferraro, Sigman, & Makse, 2019; Zhou, Mowrey, Tang, & Xu, 2015) and physics (Amini, 2010; Breskin et al., 2006; Cohen et al., 2010; Costa, 2005; da Fontoura Costa & Coelho, 2005; da Fontoura Costa & Manoel, 2003; Eckmann et al., 2007; Stepanyants & Chklovskii, 2005), serving as a promising direction to study brain criticality, neural collective dynamics, optimal neural circuitry, and the relation between brain anatomy and functions.

In the terminology of percolation, neurons are referred to as sites. The dynamic connection formed between two neurons is called the occupation of the bond between these two sites. The central question in the following percolation analysis, as suggested above, concerns the emergence of a cluster of connected sites that penetrates the brain. The brain is referred to as *percolate* if such a cluster exists. Mathematically, the criterion of being *percolate* can be defined in terms of the giant strongly connected cluster (GSCC), a special SCC whose size approaches the whole brain size in magnitude order. In Figure 3B, we demonstrate that the size, the average in-degree, and the average out-degree of the GSCC are sigmoid-like functions of *λ*. These parameters are closing to 0 when *λ* is small. Then they increase dramatically after *λ* reaches over a specific value and approximate plateau again after *λ* reaches over another specific value. This phenomenon is not accidental because it shares similarities with the observations in Figure 2A and Figure 2E. To explore the underlying mechanism, we attempt to offer an analytical characterization of the GSCC rather than limit ourselves to computational interpretations. Note that our analysis is implemented under the framework of percolation on directed graphs because the connectivity between neurons is unidirectional.

**Figure 4.**
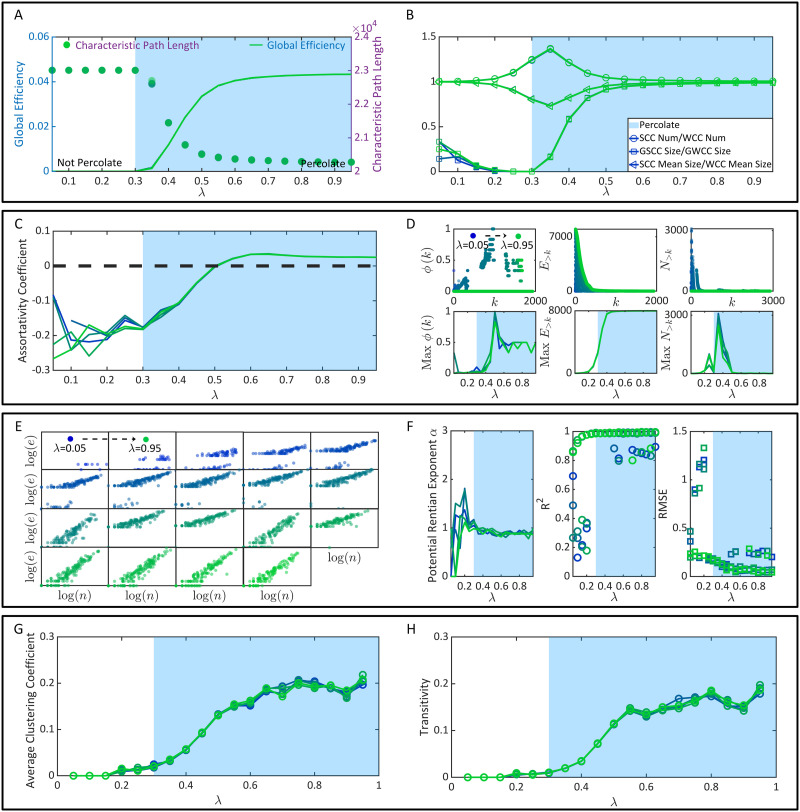
The emergence of brain function superiorities. (A) The characteristic path length and global efficiency. (B) The properties of connected clusters. (C) The assortativity coefficient. (D) The distributions (upper parallel) and the maximums of *ϕ*(*k*), *E*_>*k*_, and *N*_>*k*_ (bottom parallel). (E) Instances of (log(*n*), log(*e*)) under each *λ* condition. (F) The Rentian exponents, the RMSE, and *R*^2^. (G) The average clustering coefficient. (H) The transitivity. Note that all figures show *l* data sets of each variable independently derived from *l* (*l* = 5) times of functional connectivity generation.

Under each *λ* condition, we implement the random generation of functional connectivity *l* times (*l* = 5). Note that this setting means that all our subsequent analyses are repeated *l* times (independently repeated on every generated functional connectivity). Here we do not average results across *l* times of analyses to show that our theory is averagely consistent with experiments. On the contrary, we show *l* sets of analysis results together to suggest that the consistency between our theory and experiments as well as the consistency across the results obtained on different generated functional connectivity are not accidental (e.g., see the data points that highly overlap with each other in Figure 3D, Figure 3F, and Figure 4). Given the benefits of multiple times of functional connectivity generation, let us go back to the details of generation approach. In each time, the existence of *N*_*i*_ → *N*_*j*_ is randomized following the binomial distribution *Bi*(𝒫_*ij*_) (this is same as that in Figure 2B). Then we obtain statistics on functional connectivity, a directed graph, to calculate the probability 𝒫(*u*, *v*) for a neuron to have in-degree *u* and out-degree *v*. Based on the theory of percolation on directed graphs (Dorogovtsev et al., 2001; Li et al., 2021), the formation of the GSCC can be analyzed in terms of the probability 𝒫_*in*_ that a directed edge leads to the GSCC and the probability 𝒫_*out*_ that a directed edge comes from the GSCC. One can easily imagine that 𝒫_*in*_ and 𝒫_*out*_ increase with the size of GSCC. Analytically, 𝒫_*in*_ and 𝒫_*out*_ can be defined by their own self-consistent Equations 1 and 2. Here self-consistency means that 𝒫_*in*_ and 𝒫_*out*_ can be represented as the functions of themselves (Dorogovtsev et al., 2001; Li et al., 2021).

$${𝒫}_{\text{in}}=1-\frac{1}{\langle k\rangle }\frac{\partial }{\partial x}𝒢\left(x,y\right)\left|{}_{x=1,y=1-{𝒫}_{\text{in}}}\right.,$$ (1)

$${𝒫}_{\text{out}}=1-\frac{1}{\langle k\rangle }\frac{\partial }{\partial y}𝒢\left(x,y\right)\left|{}_{x=1-{𝒫}_{\text{out}},y=1}\right..$$ (2)

The normalization term 〈*k*〉 in Equations 1 and 2 denotes the average in-degree (or identically, the average out-degree) in Equation 3.

$$\langle k\rangle =\sum_{u,v}𝒫\left(u,v\right)u=\sum_{u,v}𝒫\left(u,v\right)v.$$ (3)

Equations 1 and 2 are derived based on the probability generating function (Equation 4), a standard and practical approach to study random graphs, especially in real data sets (Newman, Strogatz, & Watts, 2001).

$$𝒢\left(x,y\right)=\sum_{u,v}𝒫\left(u,v\right)x^{u}y^{v}.$$ (4)

Merely requiring the knowledge of 𝒫(*u*, *v*) in Equation 4, Equations 1 and 2 have been powerful enough in studying the formation of the GSCC. In other words, they can predict when the brain connectivity becomes *percolate*. Specifically, the sufficient and necessary condition for the GSCC to emerge is that Equations 1 and 2 have nontrivial solutions in (0, 1] (note that the trivial solution is 𝒫_*in*_ = 𝒫_*out*_ = 0). In practice, it is unnecessary to analytically study the nontrivial solutions of Equations 1 and 2. Instead, potential solutions can be numerically explored in a comprehensible way. Specifically, we only need to rewrite Equations 1 and 2 as functions

$${𝒲}_{\text{in}}=1-{𝒫}_{\text{in}}-\frac{1}{\langle k\rangle }\frac{\partial }{\partial x}𝒢\left(x,y\right)\left|{}_{x=1,y=1-{𝒫}_{\text{in}}}\right.,$$ (5)

$${𝒲}_{\text{out}}=1-{𝒫}_{\text{out}}-\frac{1}{\langle k\rangle }\frac{\partial }{\partial y}𝒢\left(x,y\right)\left|{}_{x=1-{𝒫}_{\text{out}},y=1}\right.,$$ (6)

and explore when 𝒲_*in*_ and 𝒲_*out*_ go through the lines 𝒲_*in*_ = 0 and 𝒲_*out*_ = 0 (see Figure 3C). When there exist non-trivial solutions ($\hat{𝒫}$_*in*_, $\hat{𝒫}$_*out*_), the probability *ϕ* that a neuron belongs to the GSCC can be calculated in Equation 7. Otherwise, the probability maintains closing to 0.

$$\phi =1-𝒢\left(1-{\hat{𝒫}}_{\text{out}},1\right)-𝒢\left(1,1-{\hat{𝒫}}_{\text{in}}\right)+𝒢\left(1-{\hat{𝒫}}_{\text{out}},1-{\hat{𝒫}}_{\text{in}}\right).$$ (7)

In Figure 3D, we compare between *ϕ* and the real probability that a neuron belongs to the GSCC in the experiment to quantify the error of our theoretical predictions. High consistency can be found between the predictions and experiment.

As can be seen in Figure 3B and Figure 3D, a phenomenon referred to as percolation transition happens at *λ* = 0.3, where the GSCC emerges suddenly (*ϕ* = 0 when *λ* ≤ 0.3 and *ϕ* ≥ 0.06 when *λ* > 0.3). This is a transition of the brain from being fragmentized to *percolate*. In other words, neurons become extensively coupled with each other to form system-level functional connectivity after *λ* reaches over 0.3. Therefore, *λ* = 0.3 serves as the percolation threshold. Moreover, probability *ϕ* in Equation 5, as well as the corresponding experimental observations, will eventually approximate to an upper bound (the growing speed approaches to 0 after *λ* reaches over 0.6). This phenomenon hints that the GSCC, reflecting functional connectivity, has a size intrinsically bounded by static connectivity. Functional connectivity forms through synaptic connections and, therefore, must be a subgraph of static connectivity. By recalculating Equations 1–5 in static connectivity, the upper bound of *ϕ* is obtained in Figure 3D and it is reached by *ϕ* when *λ* ≥ 0.6. Note intrinsic consistency between the observations in Figure 2A, Figure 2D, and Figure 2E and that in Figure 3D, even quantitative details are different as they concern about different parameters.

Furthermore, the above-observed percolation transition can also be confirmed from the perspective of diluted percolation. Under dilution condition, functional connectivity formation is not only constrained by the coactivation probability between neurons but also by the activation probability of each neuron itself. In other words, the dilution condition represents a more realistic situation where neurons are conditionally activated (e.g., by external stimulus) and functional connectivity may form only between activable neurons. The nondilution percolation analyzed above serves as a special case of the diluted one (see Figure 3E). Considering the dilution of neurons (e.g., each neuron is activated following a probability *ρ*), another version of percolation threshold *ρ*_*c*_ (the control parameter is *ρ*) can be calculated by Equations 8 and 9 (Dorogovtsev et al., 2001; Li et al., 2021)

$$\rho_{c}=\frac{\langle k\rangle }{\langle uv\rangle },$$ (8)

$$\langle uv\rangle =\sum_{u,v}𝒫\left(u,v\right)uv.$$ (9)

In Figure 3F, we can see that a meaningful *ρ*_*c*_ ∈ [0, 1] (the solution of a probability is meaningful if it is in the interval of [0, 1]) emerges only when *λ* ≥ 0.3. It decreases with *λ* until reaching to its lower bound when *λ* ≥ 0.6. The existence of a meaningful percolation threshold serves as a necessary condition for percolation transition to happen (e.g., when *λ* = 0.5, percolation transition may happen if *ρ* ≥ *ρ*_*c*_ = 0.017; when *λ* = 0.2, percolation transition never happens since *ρ*_*c*_ = 6.321 is meaningless). Therefore, the GSCC can form only after *λ* reaches over 0.3. These findings are consistent with the above nondilution percolation analysis. In Figure 3F, we directly show the potential solution of percolation threshold *ρ*_*c*_ as a function of *λ*. Later we will show the benefits of such an illustration.

In summary, our analysis presented above demonstrates that percolation, a universal formalism of criticality and phase transitions, can characterize the formation of brain functional connectivity without other top-down modeling or assumptions. All analytic calculations only require the knowledge of degree distributions in the brain connectome, which is accessible in practice. Below, we suggest that the percolation analysis alone is sufficient to explain the emergence of three key properties of brain functions.

### Percolation and Brain Function Properties

#### Percolation explains information transmission efficiency.

In the above section, we have demonstrated that functional connectivity formation can be treated as a percolation process. Starting from this section, we will study how brain function properties emerge as the characteristics of formed functional connectivity.

Let us begin with information transmission efficiency, which is manifested as the low time cost of communications between neurons or high broadcasting capacity of neural information. Such a superiority critically relies on the topological attributes of functional connectivity (Avena-Koenigsberger et al., 2018) in the real brain.

Here we attempt to quantify information transmission efficiency based on graph-theoretical metrics. Following the same idea in Figure 2B and Figure 3, we randomize functional connectivity *l* times (*l* = 5) under each *λ* condition. Given each functional connectivity, we calculate its characteristic path length (average shortest path length between all neurons) (Albert & Barabási, 2002) and global efficiency (average inverse shortest path length between all neurons) (Latora & Marchiori, 2001) (Figure 4). In general, the characteristic path length reflects the average cost of optimal information transmission (transmission always follows the minimal path length principle). The global efficiency is the average optimal information transmission efficiency. In Figure 4A, once *λ* reaches over the nondilution percolation threshold 0.3, the characteristic path length of functional connectivity drops sharply while the global efficiency increases significantly. Once *λ* reaches over 0.6, the variation speeds of these two metrics approximate 0. High consistency between these variation trends and the percolation process in Figure 3D can be observed. Meanwhile, we measure the number ratio and average size ratio between SCCs and WCCs in functional connectivity, respectively. We also calculate the size ratio between the GSCC and the GWCC (giant weakly connected cluster). These ratios are shown as functions of *λ* in Figure 4B. They principally reflect the proportion of the close-knit neural community, where information broadcasting capacity is high, within all communicable neurons (a neuron is communicable if it may communicate with at least one other neuron). In Figure 4B, the size ratio between the GSCC and the GWCC has a similar rising trend with the global efficiency, suggesting that the giant close-knit neural community will occupy more communicable neurons after percolation transition. Although the other two ratios (the number ratio and average size ratio between SCCs and WCCs) can not reflect the improvement of information broadcasting capacity by percolation transition, they fluctuate significantly near the nondilution percolation threshold and may function as observable markers of percolation transition.

In summary, information transmission efficiency improvement is demonstrated as a byproduct of the percolation process. Percolation transition may be a critical condition for high transmission efficiency to emerge.

#### Percolation explains robust flexibility.

Then we turn to study the robust flexibility of brain connectivity, which is the capacity of the brain to tolerate the large-scale loss of neurons or synaptic connections (e.g., by lesions (Aerts et al., 2016; Joyce et al., 2013; Kaiser et al., 2007)) while maintaining robust brain functions.

In general, robust flexibility can be studied directly and indirectly (Rubinov & Sporns, 2010b). Direct analysis of robust flexibility usually compares functional connectivity before and after presumed attacks (e.g., removing some neurons) (Aerts et al., 2016; Joyce et al., 2013; Kaiser et al., 2007). We suggest that these attacks, no matter if they are random or targeted, are equivalent to the dilution introduced in diluted percolation analysis (see Figure 3E–F). Those attacked neurons or directionally adjacent relations (DA) are never included in functional connectivity and, therefore, can be treated as diluted. From this perspective, one can understand the motivation underlying the direct illustration of dilution percolation threshold *ρ*_*c*_ as a function of *λ* in Figure 3F. It benefits our analysis by showing the maximum tolerable attack intensity 1 − *ρ*_*c*_ of the brain while maintaining *percolate*. Based on Figure 3F, we discover that the brain can not tolerate attacks until *λ* reaches over 0.3. The robust flexibility increases sharply until *λ* reaches over 0.6, after which the increasing speed becomes stable.

As for the indirect analysis of robust flexibility, we implement it in terms of the assortativity coefficient (Newman, 2002) and the rich-club coefficient (Ball et al., 2014; Colizza, Flammini, Serrano, & Vespignani, 2006; Van Den Heuvel & Sporns, 2011). The assortativity coefficient is the Pearson correlation between the degrees of all neurons on two opposite ends of a DA. Brain connectivity with a positive assortativity coefficient may have a comparatively robust community of mutually interconnected high-degree hubs, while brain connectivity with a negative assortativity coefficient may have widely distributed and vulnerable high-degree hubs (Rubinov & Sporns, 2010b). Similarly, the rich club coefficient *ϕ*(*k*) is the number ratio of the DAs between neurons of degree > *k* (denoted by *E*_>*k*_), when all neurons of degree ≤ *k* have been removed, to the maximum DAs that such neurons can share (denoted by *N*_>*k*_) (Ball et al., 2014; Colizza et al., 2006; Van Den Heuvel & Sporns, 2011). The calculations of these two metrics can be implemented utilizing a toolbox designed by Rubinov and Sporns (2010b). In Figure 4C, we discover that the assortativity coefficient significantly increases once *λ* reaches over 0.3. It becomes positive after *λ* reaches over 0.5 and becomes relatively changeless after *λ* reaches over 0.6. Similar variation trends can also be observed in the maximum *ϕ*(*k*) (the maximum value is obtained through the comparison across different *k*). A slight difference lies in that the maximum *ϕ*(*k*) reaches its peak when *λ* = 0.5 and then drops until *λ* = 0.6 (Figure 4D).

In sum, the robust flexibility of functional connectivity, no matter if it is analyzed directly or indirectly, experiences a sharp increase after percolation transition (*λ* = 0.3). Moreover, it is again intrinsically limited by static connectivity and reaches its bound after *λ* ≥ 0.6.

#### Percolation explains brain economy.

Finally, we analyze brain economy from the perspectives of network wiring and network running, accordingly.

Measuring the physical embedding efficiency of functional connectivity is a promising approach to analyze network wiring economy (Bassett et al., 2010). A key organizational principle shared by various physical information processing systems is the isometric scaling relationship between the number of processing units (e.g., neurons) and the number of connections (e.g., directionally adjacent relations), known as the Rentian scaling. Such a property reveals the relation between the dimensionality of system topology and information processing capacity. In general, the Rentian scaling corresponds to an economic wiring paradigm of embedding a high-dimensional functional topology in a low-dimensional physical space (Bassett et al., 2010; Chen, 1999; Ozaktas, 1992). To verify the existence of the Rentian scaling, we need to partition the physical space into *m* cubes. Then we count the number *n* of neurons within each cube and the number *e* of directionally adjacent relations (DAs) crossing the cube boundaries. The Rentian scaling exists if there is a statistically significant linear regression relation log(*e*) = *α* log(*n*) + *β* and *α* ∈ (0, 1). Here *α* is referred to as the physical Rentian exponent. A smaller significant exponent corresponds to higher efficiency. We use the coordinate information of neurons to embed functional connectivity into real size physical space, where the partition number is set as *m* = 300. In Figure 4E, we show instances of (log(*n*), log(*e*)) distributions generated from functional connectivity under each *λ* condition. Qualitatively, we can already find that (log(*n*), log(*e*)) may not follow a significant linear regression relation when *λ* is small. Quantitatively, we discover that the linear regression performance before the percolation transition, *λ* = 0.3, is weak and unstable. This phenomenon is expectable because system-level functional connectivity has not emerged yet, and the physical space is occupied by isolated and inefficient neurons (see Figure 4F). Once *λ* approaches and further reaches over 0.3, the linear regression becomes significant with an average physical Rentian exponent *α* ∼ 0.8999 (the standard error is ∼0.0464). A slight decrease of *α* can be observed when *λ* ∈ (0.3, 0.6), suggesting that the physical embedding becomes relatively more efficient. Except being reported alone, the physical Rentian exponent *α* calculated through physical space partition can also be compared to its theoretical minimum to draw the same conclusion from another perspective (e.g., see Bassett et al., 2010).

As for the economy of network running, it should be noted that a possible misconception about the metabolic economy is that the metabolic economy vanishes after system-level functional connectivity emerges. Here we need to emphasize the inequivalence between high metabolic cost and low metabolic economy. System-level functional connectivity with high metabolic cost (massive neural dynamics and high E/I balance are metabolically costly; Barta & Kostal, 2019; Bullmore & Sporns, 2012) is not necessarily inefficient if it can support large amounts of functions. Low efficiency corresponds to high costs but low functional capacity. In real brains, high metabolic consumption is inevitable considering billions of neurons and synapses (Bullmore & Sporns, 2012); metabolic economy is mainly determined by the functional capacity payoff. Such a payoff in real brains is usually shaped by functional segregation and integration capacities (Rubinov & Sporns, 2010b). Specifically, functional segregation is manifested as specialized and distributed information processing in densely connected groups of neural clusters, which can be analyzed in terms of modular structure (Rubinov & Sporns, 2010b) and quantified applying the clustering coefficient (Fagiolo, 2007; Watts & Strogatz, 1998) and the transitivity (Newman, 2003). In Figure 4G–H, these two metrics are shown as functions of *λ*, respectively. While they increase sharply after percolation transition, their increase rates drop once *λ* reaches over 0.5. In other words, modular structures experience massive mergers after percolation transition, becoming larger and more close-knit. However, the ever-rising *λ* > 0.5 can not improve functional segregation without limitation. These modular structures in functional connectivity are ultimately restricted by static connectivity. As for functional integration, it refers to the ability to collect and combine specialized information from distributed neural clusters (Rubinov & Sporns, 2010b). The characteristic path length and the global efficiency shown in Figure 4A are practical metrics of this property, which have been demonstrated as explicable by percolation.

Combine the information in Figure 4E–H, we can see that both network wiring economy and network running economy are optimized after *λ* ≥ 0.3. Considering that every rise in the E/I balance causes higher energy consumption (Barta & Kostal, 2019; Bullmore & Sporns, 2012) but may not bring additional payoffs (e.g., network running economy does not significantly increase after *λ* ≥ 0.5), we suggest that *λ* ∼ 0.5 may be an optimal choice for the brain to maintain economy.

## DISCUSSION

### The Optimal Synaptic E/I Balance for Brain Functions

Let us move forward our analysis by incorporating all above presented findings to solve a critical question concerned in neuroscience: what is the optimal synaptic E/I balance?

There are several vital values of the E/I balance *λ* according to our previous analysis: the nondilution percolation threshold *λ* = 0.3 (E/I balance is 3:7) where percolation transition happens; the approximate value *λ* = 0.5 (E/I balance is 1:1) which reconciles the actual size of the GSCC with its increasing rates; the approximate value *λ* = 0.6 (E/I balance is 3:2), after which percolation approximates to its bound. The advantageous characteristics of brain functions, including efficiency (Avena-Koenigsberger et al., 2018), robustness (Aerts et al., 2016; Joyce et al., 2013; Kaiser et al., 2007), and economy (Bullmore & Sporns, 2012), principally experience sharp increases after percolation transition at *λ* = 0.3 and become changeless after *λ* = 0.6 because they are intrinsically bounded by the characteristics of static or anatomical connectivity. Information transmission efficiency, robust flexibility, and network wiring economy have relatively large actual quantities and high increasing speeds near *λ* = 0.5, after which their increasing speeds gradually approximate 0 and are not sufficient to act as payoffs of the rising of *λ*. Therefore, the actual value of network running economy is very likely optimized near *λ* = 0.5. Above this value a sharp drop is observed. Moreover, a significantly high E/I balance may damage information encoding efficiency (Barta & Kostal, 2019; Sprekeler, 2017) and membrane potential stabilizing (Sadeh & Clopath, 2021; Sprekeler, 2017). Taking all these pieces of evidence together, it is suggested that an optimal E/I balance for the brain to guarantee advantageous properties simultaneously may be *λ* ∼ 0.5, consistent with the previous in vitro experimental finding (Shew et al., 2011). Furthermore, this inferred optimal E/I balance by percolation theory corroborates the findings of the sufficient condition for neural dynamics in the brain to be critical (Poil, Hardstone, Mansvelder, & Linkenkaer-Hansen, 2012). In other words, it provides explanations of the origin of cortical criticality, a widespread phenomenon in multiple species’ brains (Beggs & Timme, 2012; Chialvo, 2004; Fontenele et al., 2019; Fosque, Williams-García, Beggs, & Ortiz, 2021; Gautam, Hoang, McClanahan, Grady, & Shew, 2015; Millman, Mihalas, Kirkwood, & Niebur, 2010; Williams-García, Moore, Beggs, & Ortiz, 2014), from a new perspective. In our research, the E/I balance *λ* is defined as the fraction of excitatory synapses in all synapses. Because the excitation/inhibition strength in Equation 10 is uniformly generated, the predicted optimal E/I balance *λ* ∼ 0.5 implies a balance between excitation *W*_*E*_ and inhibition *W*_*I*_ strengths on the whole brain. This result is consistent with a well-known finding that neural activities with high and robust entropy occur when *W*_*E*_ and *W*_*I*_ are balanced (Agrawal et al., 2018). Meanwhile, we can further transform the optimal E/I balance *λ* ∼ 0.5 to parameter *ψ*, the fraction of excitatory neurons in all neurons, since *W*_*E*_, *W*_*I*_, and *ψ* are mathematically related (Agrawal et al., 2018). To guarantee high and robust entropy, a large *ψ* can be derived according to Agrawal et al. (2018). These results corroborate the abundance of excitatory neurons in mammalian cortices (Hendry, Schwark, Jones, & Yan, 1987; Meinecke & Peters, 1987; Sahara, Yanagawa, O’Leary, & Stevens, 2012).

A valuable direction of future exploration may be generalizing the quantification of efficiency, robustness, and economy to verify whether the optimal E/I balance that simultaneously guarantees these advantageous properties still matches percolation theory predictions. The generalization is necessary since the measurements of efficiency, robustness, and economy in brains remains as open challenges. For instance, the graph-theoretical metrics considered in information transmission efficiency analysis (e.g., global efficiency) could be nonideal because neural signal communication may not exhibit near-minimal path length characteristics (Avena-Koenigsberger et al., 2018; Goñi et al., 2014). Alternative metrics (Avena-Koenigsberger et al., 2018; Seguin, Tian, & Zalesky, 2020; Seguin, Van Den Heuvel, & Zalesky, 2018) have been recently developed and can be applied to reflect transmission efficiency more appropriately.

### Percolation Theory of the Brain: Opportunities and Challenges

In the present study, we have suggested percolation as a window to understand how brain function properties emerge during functional connectivity formation. The congruent relationship between brain connectivity and percolation is natural and comprehensible. Just like the porous stone immersed in a pool of water, the brain is “porous” in terms of neurons and immersed in a “pool” of information. Through simple analytical calculations, percolation analysis has shown strong consistency both in theoretical and experimental observations. Perhaps because of these advantages, percolation theory has attracted emerging interest in neuroscience. From early explorations that combine limited neural morphology data with computational simulations to analyze percolation (Costa, 2005; da Fontoura Costa & Coelho, 2005; da Fontoura Costa & Manoel, 2003; Stepanyants & Chklovskii, 2005) to more recent works that study percolation directly on the relatively small-scale and coarse-grained brain connectome and electrically stimulated neural dynamics data captured from living neural networks (e.g., primary neural cultures in rat hippocampus) (Amini, 2010; Breskin et al., 2006; Cohen et al., 2010; Eckmann et al., 2007), the efforts from physics have inspired numerous follow-up explorations in neuroscience (Bordier et al., 2017; Carvalho et al., 2020; Del Ferraro et al., 2018; Kozma & Puljic, 2015; Lucini et al., 2019; Zhou et al., 2015). These studies capture an elegant and enlightening view about optimal neural circuitry, neural collective dynamics, criticality, and the relation between brain connectivity and brain functions. Built on these works, we present a more systematic and biologically justified framework to formalize functional connectivity formation as percolation on random directed graphs. The merit of our framework is fourfold. First, different from the bootstrap (e.g., see Eckmann et al., 2007) or quorum (e.g., see Cohen et al., 2010) percolation analysis in previous studies, we leave the criterion of occupation as a work of neural dynamics computation rather than preset the occupation profile of neurons. In the future, this criterion can be optimized by synchronous neural dynamics recording when the required technology matures. Second, percolation on directed graphs can capture the directionality of neural dynamics diffusion. Third, the percolation defined on random graphs is a natural reflection of the inequivalence between static and functional connectivity, consistent with the fact that static connectivity serves as a platform of the dynamic one. Finally, we distinguish between dilution and nondilution conditions, enabling us to characterize functional connectivity formation in stimulus-driven or attacked situations. These properties enhance the capacity of our framework to accommodate the real characteristics of brain connectivity.

Built on the equivalence between functional connectivity formation and the percolation controlled by synaptic E/I balance, we reveal that brain function properties emerge as byproducts of percolation transition. Although the present percolation framework is universal, we hypothesize that the actual quantities of percolation threshold, the optimal E/I balance, and the E/I balance after which these properties approximate to their bounds determined by static connectivity, may vary across different species, ages, cortical states, and even be able to serve as biomarkers of nerve diseases. This speculation is well-founded because the E/I balance oscillates daily (Bridi et al., 2020; Tao, Li, & Zhang, 2014) and may change owing to dysfunction (Grent et al., 2018; Ramírez-Toraño et al., 2021). These changes may be consequences of specific variations of percolation attributes, which remain for future exploration.

### Understanding Brain Function Characteristics Through Physics

Rooted in emergentism, the present study attempts to explore the origin mechanisms of brain function characteristics from a physics perspective and avoid assumption-based modeling. The underlying mechanisms are suggested to be described by percolation, a universal characterization of critical phenomena and phase transitions (Dorogovtsev et al., 2001; Li et al., 2021). Our experiments in the largest yet brain connectome of the fruit fly, *Drosophila melanogaster* (Pipkin, 2020; Schlegel et al., 2021; Xu et al., 2020), have demonstrated the capacity of percolation to explain the formation of functional connectivity and the emergence of brain function properties without additional assumptions. The strong explanatory power of percolation theory toward these neuroscience concepts is experimentally observed, even though they are initially studied in different scientific fields. Such an intriguing connection reemphasizes the nature of the brain as a physical system. Recently, this physics perspective has seen substantial progress in neuroscience. For instance, the free-energy principle is demonstrated as a unified foundation of perception, action, and learning (Friston, 2009, 2010, 2012; Friston & Kiebel, 2009; Guevara, 2021). Cortical criticality is discovered to account for the efficient transformation between cortical states (Beggs & Timme, 2012; Chialvo, 2004; Fontenele et al., 2019; Fosque et al., 2021; Gautam et al., 2015; Millman et al., 2010; Williams-García et al., 2014). Moreover, information thermodynamics is revealed as a bridge between the physical and the informational brain (Capolupo, Freeman, & Vitiello, 2013; Collell & Fauquet, 2015; Sartori, Granger, Lee, & Horowitz, 2014; Sengupta, Stemmler, & Friston, 2013; Street, 2020). In the future, this rising direction may continue contributing to neuroscience as a window to understand the intrinsic bases of brain function characteristics.

To conclude, the parallels discovered between functional connectivity formation and the percolation on random directed graphs controlled by synaptic E/I balance serve as a new pathway toward understanding the emergence of brain function characteristics. The percolation, an elegant formalism of critical phenomena and phase transitions, may elucidate the emergence of brain function characters from brain connectivity.

## MATERIALS AND METHODS

### Brain Connectome Acquisition

The brain connectome data is released as a part of the FlyEM project (Xu et al., 2020). The connectome imaging is implemented applying the focused ion beam scanning electron microscopy (FIB-SEM) technology. Then images are cleaned and enhanced by a series of pipelines (e.g., automated flood-filling network segmentation), generating the most fine-grained and high-throughput connectome of the fruit fly central brain (∼2.5 × 10^5^ nm in each dimension) to date (Xu et al., 2020). There are 21,662 traced (all main branches within the volume are reconstructed) and uncropped (main arbors are contained in the volume) neurons as well as 4,495 traced, cropped, and large (≥1,000 synaptic connections) neurons in the connectome. The complex wiring diagram between neurons consists of ∼6 × 10^6^ traced and uncropped presynaptic sites as well as ∼1.8 × 10^7^ traced and uncropped post-synaptic densities (Xu et al., 2020).

To efficiently acquire and handle such a large dataset, Google has designed a system *neu*Print to organize brain connectome in an accessible manner. Readers can follow the instructions in Xu et al. (2020) to download and process the brain connectome data in *neu*Print. Although we acquire the whole brain connectome, we include neurons and synapses in analyses only when the cell bodies are positioned precisely (assigned with a 10-nm spatial resolution coordinate). The coordinate information is required expressly because it is necessary in connectivity visualization and the Rentian scaling analysis (Bassett et al., 2010; Chen, 1999; Ozaktas, 1992). The selected data set contains 23,008 neurons, 4,967,364 synaptic connections (synaptic clefts), and 63,5761 pairs of directionally adjacent relations (two neurons are directionally adjacent if one synaptic connection comes out of one neuron and leads to another).

### Variables in Brain Connectome Description

To present a systematic characterization of brain connectome, we consider two informative scales: macroscopic scale (brain regions) and microscopic scales (neurons).

On macroscopic scale, the parcellation of brain regions has been provided by Xu et al. (2020). According to the upstream and downstream information of each synapse, we can describe the macroscopic static connectivity with the variables given in Table 3.

**Figure 5.**
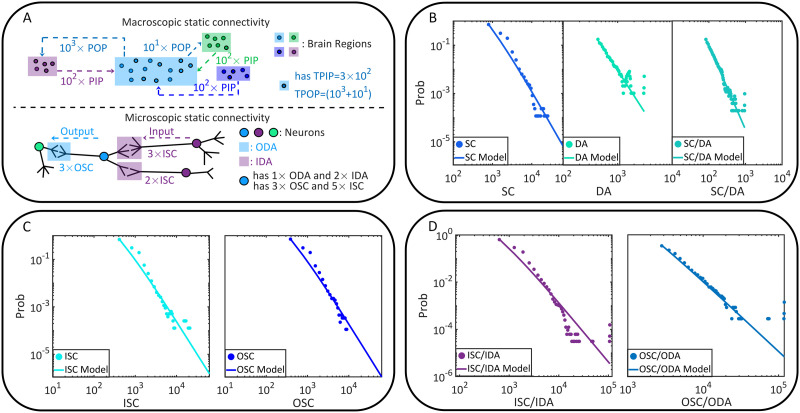
Supplementary information of static connectivity characterization. (A) Supplementary variables of macroscopic static connectivity and microscopic static connectivity are graphically illustrated in an instance. (B) The power law models of synaptic connection number (SC), directionally adjacent relation number (DA), and the number of synaptic connections per directionally adjacent relation (SC/DA). (C) The power law models of input synaptic connection number (ISC) and output synaptic connection number (OSC). (D) The power law models of the number of synaptic connections between one neuron and one of its presynaptic neuron (ISC/IDA) and the number of synaptic connections between one neuron and one of its postsynaptic neuron (OSC/ODA).

One can reasonably treat the macroscopic static connectivity as a directed graph. Nodes are brain regions and there may be multiple directed edges (PIP and POP) between each pair of nodes. By summing up all PIPs or POPs of a brain region, we can measure the macroscopic in-degree (TPIP) and out-degree (TPOP) of it. In our research, variables TPIP and TPOP are used in our main analyses (please see Tables 1 and 2 and Figure 1 in the main text) while variables PIP and POP are used to provide additional information (please see Tables 5 and 6 and Figure 5).

Similarly, we can define the microscopic static connectivity by the variables given in Table 4.

**Table 4.** Microscopic variable definitions

Variable	Meaning
SC	All synaptic connections featured by one neuron
ISC	All synaptic connections received by one neuron from other neurons
OSC	All synaptic connections coming from one neuron to other neurons
DA	All directionally adjacent relations between one neuron and other neurons
IDA	All directionally adjacent relations between one neuron and its presynaptic neurons
ODA	All directionally adjacent relations between one neuron and its postsynaptic neurons
SC/DA	Synaptic connections between one neuron and one of its adjacent neuron (the number of SCs per DA)
ISC/IDA	Synaptic connections between one neuron and one of its presynaptic neuron (the number of ISCs per IDA)
OSC/ODA	Synaptic connections between one neuron and one of its postsynaptic neuron (the number of OSCs per ODA)

The microscopic static connectivity corresponds to a directed graph where nodes are neurons. With a fine granularity, we can treat synaptic connections as directed edges. Thus, there may be multiple edges (SCs) between a selected neuron and one of its adjacent neurons. We can further tell whether these edges are coming from (OSCs) or leading to (ISCs) this selected neuron. With a coarse granularity, we can treat directionally adjacent relations as directed edges. Under this condition, there is only one edge (DA) between two adjacent neurons and we can similarly distinguish between IDAs and ODAs. The fine-grained information is necessary in neural dynamics computation while the coarse-grained information in useful in analyzing microscopic functional connectivity. Therefore, we also calculate SC/DA, ISC/IDA, and OSC/ODA to reflect how a neuron allocates synaptic connections to each of its neighbors. These variables offer mappings between fine-grained and coarse-grained information. In our research, variables relevant with DA are used in our main analyses (please see Tables 1 and 2 and Figure 1 in the main text) while other variables are used to provide auxiliary information (please see Tables 5 and 6 and Figure 5).

**Table 5.** Power law analysis results (supplementary information)

Type	Variable	Probability distribution	Goodness of estimation	Scale-free or not
Microscopic	SC	𝒫 (SC = *n*) ∝ *n*^−2.87^	0.0104	Yes
Microscopic	ISC	𝒫 (ISC = *n*) ∝ *n*^−2.78^	0.0177	Yes
Microscopic	OSC	𝒫 (OSC = *n*) ∝ *n*^−2.81^	0.0172	Yes
Microscopic	DA	𝒫 (DA = *n*) ∝ *n*^−3.53^	0.0248	No
Microscopic	SC/DA	𝒫 (SC/DA = *n*) ∝ *n*^−3.49^	0.0244	No
Microscopic	ISC/IDA	𝒫 (ISC/IDA = *n*) ∝ *n*^−2.53^	0.0211	Yes
Microscopic	OSC/ODA	𝒫 (OSC/ODA = *n*) ∝ *n*^−2.99^	0.0183	Yes

*Note*. Being scale-free requires 𝒫 ∝ *n*^−*α*^, where *α* ∈ (2, 3). Goodness of estimation is expected as to be less than 0.05.

**Table 6.** Symmetry analysis results (supplementary information)

Variable	Variable	Pearson correlation	*p*	Average change fraction	Symmetric degree
PIP	POP	0.9833	< 10^−10^	$\frac{\left|\text{PIP}-\text{POP}\right|}{\text{POP}}$ = 0.6176	Strictly strong
ISC	OSC	0.8977	< 10^−10^	$\frac{\left|\text{ISC}-\text{OSC}\right|}{\text{OSC}}$ = 1.7042	Relatively weak
ISC/IDA	OSC/ODA	0.8164	< 10^−10^	$\frac{\left|\text{ISC}/\text{IDA}-\text{OSC}/\text{ODA}\right|}{\text{OSC}/\text{ODA}}$ = 0.8649	Less strong

*Note*. Strong symmetry requires a strong positive correlation (e.g., correlation > 0.9 and *p* < 10^−3^). Strong symmetry implies a small average change fraction (e.g., fraction < 1). The term “strictly strong” means that the strictest criterion of strong symmetry is completely satisfied. The term “less strong” means that the strictest criterion of strong symmetry is partly satisfied. The term “relatively weak” means that the strictest criterion of strong symmetry is not satisfied.

### Power Law Analysis and Symmetry Analysis

In our research, power-law analysis in binned empirical data is implemented applying an open-source toolbox (R. Virkar & Clauset, 2014). Corresponding mathematics derivations can be seen in Y. Virkar and Clauset (2014). We first do binning on variables of interest, where the number of linearly spaced bins is set as 150. In real cases, the power law may only hold on specific tails {𝒫(*A*)|*A* ≥ *A*′} of the empirical distribution of variable *A*, where *A*′ denotes a certain distribution cutoff (Clauset, Shalizi, & Newman, 2009). We apply the Kolmogorov-Smirnov-statistic-based approach (Y. Virkar & Clauset, 2014) to estimate these distribution cutoffs. Then we implement a maximum likelihood estimation of power-law exponents (Y. Virkar & Clauset, 2014) on the distribution tails above cutoffs. Corresponding Kolmogorov–Smirnov statistics can be calculated between the cumulative probability distributions of estimated power law models and empirical cumulative probability distributions to reflect the goodness of estimation (Y. Virkar & Clauset, 2014). An ideal estimation is expected to has a sufficiently small Kolmogorov–Smirnov statistic (<0.05 is suggested as a strict criterion). The authors of Y. Virkar and Clauset (2014) also propose a statistic significance test based on Kolmogorov–Smirnov statistics. Although the test measures the statistic significance of rejecting the power law hypothesis, it cannot further imply the correctness of the estimated power law model (Y. Virkar & Clauset, 2014). Therefore, the test is unnecessary if Kolmogorov–Smirnov statistics have been sufficiently small.

In Table 5 and Figure 5, we present the auxiliary results of power law analysis. These results are derived on supplementary variables in static brain connectivity descriptions and provide corroborative evidence for the main text. In general, macroscopic static connectivity is suggested as plausibly scale-free. However, the potential scale-free property of microscopic static connectivity seems to be uncertain and nonrobust.

The symmetry analysis for static connectivity is implemented based on Pearson correlation and average change fraction between input-related variables and output-related variables. Pearson correlation reflects if output-related variables have similar variation trends with input-related variables. Average change fraction of output-related variables compared with input-related variables reflects if these two kinds of variables are quantitatively similar. The symmetric static connectivity is expected to have balanced input-related and output-related variables and, therefore, is required to feature significant positive correlation (e.g., larger than 0.9) and small average change fraction (e.g., smaller than 1). We refer to these two requirements as the strictest criterion of strong symmetry. To present a more accurate analysis, we suggest that three levels of symmetry can be specified. The symmetry is suggested as strictly strong if the strictest criterion is completely satisfied. If the criterion is partly satisfied, the symmetry is treated as less strong. The symmetry is suggested as relatively weak if both requirements are not satisfied (please note that this case does not imply that static connectivity is purely asymmetric, it only suggests the existence of potential local asymmetry).

In Table 6, we present the symmetry analysis results of supplementary variables in static brain connectivity descriptions. These results serve as pieces of corroborative evidence to support our findings in the main text. In general, macroscopic static connectivity is suggested as robustly symmetric. Although microscopic static connectivity is approximately symmetric, its symmetric degree is not as strong as the macroscopic one.

### Coactivation Probability Calculation

Here we introduce the method of functional connectivity generation. Specifically, every directionally adjacent relation (DA) *N*_*i*_ → *N*_*j*_ (here *N*_*i*_ and *N*_*j*_ are neurons) has a static connection strength *S*_*ij*_ defined by Equation 10. In the definition, random variable *X*_*i*_ ∈ {0, 1} determines whether *N*_*i*_ is an excitatory or inhibitory neuron. Random variables *Y*_*ij*_ ∼ 𝒰_(0,1]_ and *Z*_*ij*_ ∈ ∼ 𝒰_[−1,0]_ are generated uniformly to measure excitation/inhibition strength (e.g., conductance). Notion *α*_*ij*_ denotes the number of synaptic connections (SC) from *N*_*i*_ to *N*_*j*_ (SC/DA, the precise number of SCs per DA) and 〈·〉 denotes the expectation. By calculating $\frac{\alpha_{ij}}{{\langle \alpha_{ij}\rangle }_{i,j}}$ in Equation 10, we can quantify the effects of SCs on *S*_*ij*_.

$$S_{ij}=\frac{\alpha_{ij}}{{\langle \alpha_{ij}\rangle }_{i,j}}\left[X_{i}Y_{ij}+\left(1-X_{i}\right)Z_{ij}\right].$$ (10)

Under each condition of E/I balance *λ*, a set of variable {*X*_1_, …, *X*_*n*_} (here *n* denotes the number of neurons) is randomly initialized. Then, we update {*X*_1_, …, *X*_*n*_} by the following algorithm: (1) measure $\hat{\lambda }$, the fraction of excitatory SCs in all SCs based on the current configuration of {*X*_1_, …, *X*_*n*_}, and calculate the difference |$\hat{\lambda }$ − *λ*|; (2) randomly select a neuron *N*_*i*_ and change the corresponding *X*_*i*_ (e.g., change *X*_*i*_ from 1 to 0 or from 0 to 1). (the change will not be kept unless it reduces |$\hat{\lambda }$ − *λ*|); (3) repeat steps (1 and 2) until $\hat{\lambda }$ ∼ *λ*. Based on this algorithm, the fraction of excitatory synapses in all synapses is principally controlled by *λ* with reasonable errors in practice.

Then, we measure coactivation probability 𝒫_*ij*_ in Equation 11 to define the dynamic connection strength, where *δ*(·) denotes the Dirac delta function. Probability 𝒫_*ij*_ is quantified in term of that an activated *N*_*i*_ can activate *N*_*j*_ in *m* times of experiments (*m* = 100).

$${𝒫}_{ij}=\frac{1}{m}\sum_{k=1}^{m}\delta \left(I_{i}^{k}-I_{j}^{k}\right).$$ (11)

Each time all neurons are initialized at the resting potential, −70 mV. Then we continuously activate *N*_*i*_ (make $I_{i}^{k}$ = 1) and several other randomly selected presynaptic neurons of *N*_*j*_ during an interval of [0, *t*] (we define *t* = 50 ms). We ensure that at least 20% of presynaptic neurons of *N*_*j*_ are activated. Such activation intensity is no longer accidental and should be strong enough to affect *N*_*j*_ in the real brain. Any activation will be marked by Equation 12, where $I_{j}^{k}$ = 1 if *N*_*j*_ is activated at least one time during [0, *t*], otherwise $I_{j}^{k}$ = 0. The criterion of activation in Equation 12 is to verify if the actual membrane potential *V*_*j*_(*τ*) reaches the spiking threshold $\hat{V}$ = −50 mV based on the unit step function *ν*(·).

$$I_{j}^{k}=\nu \left[\sum_{\tau =0}^{t}\nu \left(V_{\tau }-\hat{V}\right)\right].$$ (12)

We calculate the actual membrane potential *V*_*j*_(*τ*) applying the standard leaky integrate-and-fire (LIF) model (Gerstner et al., 2014) in Equation 13, where *τ*_*m*_ is the membrane time constant (*τ*_*m*_ = 10 ms), notion *N*_*h*_ searches through all presynaptic neurons of *N*_*j*_, and $\tau_{h}^{f}$ represents the arrival time of *f*-th spike of neuron *N*_*h*_.

$$\frac{\partial V_{j}\left(\tau \right)}{\partial \tau }=-\frac{1}{\tau_{m}}\left[V_{j}\left(\tau \right)-\hat{V}\right]-\sum_{N_{h}}S_{hj}\left\{\sum_{f}\delta \left(\tau -\tau_{h}^{f}\right)\left[V_{j}\left(\tau_{h}^{f}\right)-V_{h}\left(\tau_{h}^{f}\right)\right]\right\}.$$ (13)

To make such a computationally costly experiment accessible for readers, we release our results in Tian and Sun (2021). Readers can freely download the dataset to obtain the co-activation probability 𝒫_*ij*_ under each *λ* condition.

### Quantification of Efficiency, Robustness, and Economy

In our research, the measurements of the characteristic path length (Albert & Barabási, 2002), the global efficiency (Latora & Marchiori, 2001), the assortativity coefficient (Newman, 2002), the rich-club coefficient (Ball et al., 2014; Colizza et al., 2006; Van Den Heuvel & Sporns, 2011), the physical Rentian exponent (Bassett et al., 2010; Chen, 1999; Ozaktas, 1992), the clustering coefficient (Watts & Strogatz, 1998), and the transitivity (Newman, 2003) are implemented based on the toolbox released by Rubinov and Sporns (2010b). This widely used toolbox and its instructions can be found in Rubinov and Sporns (2010a). Readers can use the adjacent matrix of functional connectivity as an input to calculate these properties. One thing to note is that the original version of the toolbox defines the graph distance between two unconnected neurons (there is no path between them) as infinity. Although this definition is mathematically justified, it may bring inconvenience in computational implementations because inf usually leads to inf or nan in computer programs. Common practices to avoid the influence of potential infinite values on target results are either excluding these infinite values during calculations or replacing them with *n* + 1 (here *n* denotes the total number of neurons). To maintain the sample size in analyses, our research chooses the second approach.

## ACKNOWLEDGMENTS

Authors are grateful for discussions and assistance of Drs. Ziyang Zhang and Yaoyuan Wang from the Laboratory of Advanced Computing and Storage, Central Research Institute, 2012 Laboratories, Huawei Technologies Co. Ltd., Beijing, 100084, China.

## AUTHOR CONTRIBUTIONS

Yang Tian: Conceptualization; Data curation; Formal analysis; Investigation; Methodology; Project administration; Software; Validation; Visualization; Writing – original draft. Pei Sun: Conceptualization; Funding acquisition; Investigation; Project administration; Resources; Supervision; Validation; Writing – review & editing.

## FUNDING INFORMATION

Pei Sun, Artificial and General Intelligence Research Program of Guo Qiang Research Institute at Tsinghua University, Award ID: 2020GQG1017.

## TECHNICAL TERMS

Functional connectivity: The connectivity formed by dynamic interactions among neurons.
Static connectivity: The anatomic connectivity of the brain, irrespective of dynamic interactions among neurons.
Phase transition: The physical process of transition between system states defined by some control parameters.
Percolation transition: The transition between system connectivity states (e.g., from being fragmentized to being percolate).
Weakly connected cluster: A subgraph of a directed graph, where each node is an endpoint of at least one directed edge coming to it or one directed edge coming out from it.
Strongly connected cluster: A subgraph of a directed graph, where if any two nodes are connected by a directed path (e.g., coming from the first node to the second one), then there must exist another antidromic directed path between them (e.g., coming from the second node to the first one).
Percolation on random graphs: A kind of percolation process defined on random graphs, concerning the connectivity states between nodes (referred to as sites in the terminology of percolation) or edges (referred to as bonds in the terminology of percolation).
Percolation process: An evolutionary process of system connectivity states controlled by specific parameters, where each system unit is randomly occupied and system connectivity states are characterized by the connections of occupied system units.
Percolation theory: A physics theory that characterizes critical phenomena and phase transitions from a probabilistic and geometric perspective.
